# A DNA nanostructure‐Hif‐1α inducer complex as novel nanotherapy against cisplatin‐induced acute kidney injury

**DOI:** 10.1111/cpr.13601

**Published:** 2024-01-14

**Authors:** Yuanchong Chen, Jiangshan Xu, Sirong Shi, Wenjuan Ma, Weitong Cui, Ran Yan, Yunfeng Lin

**Affiliations:** ^1^ State Key Laboratory of Oral Diseases, National Center for Stomatology, National Clinical Research Center for Oral Diseases, West China Hospital of Stomatology Sichuan University Chengdu Sichuan China; ^2^ Sichuan Provincial Engineering Research Center of Oral Biomaterials Chengdu Sichuan China

## Abstract

Since its discovery in 1978, cisplatin‐based chemotherapy regimens have served a pivotal role in human cancer treatment, saving millions of lives. However, its high risk still poses a significant challenge for cisplatin‐induced acute kidney injury (AKI), which occurs in 30% of cisplatin‐treated patients. Unfortunately, no effective solution for preventing or managing this severe complication, which greatly impacts its clinical administration. Kidney is the main organ injured by cisplatin, and the injury is related to cisplatin‐induced cell apoptosis and DNA injury. Therefore, to achieve the safe use of cisplatin in tumour treatment, the key lies in identifying a kidney treatment that can effectively minimize cisplatin nephrotoxicity. Here, we successfully synthesized and applied a DNA‐nanostructure complex, named TFG, which contains tetrahedral framework nucleic acids (tFNAs) and FG‐4592, a novel Hif‐1α inducer. As cargo, TFG is composed entirely of DNA strands. It possesses low nephrotoxicity and renal aggregation properties while FG‐4592 is able to relieve renal injury by downregulating the apoptosis signal pathways. And it can relieve cisplatin‐induced renal injury when taken cisplatin treatment. This work aims to enhance chemotherapy protection in tumour patients by using TFG, a DNA‐based nanomedicines to kidney. This work has the potential to revolutionize the treatment of renal diseases, particularly drug‐induced kidney injury, leading to improved clinical outcomes.

## Graphical abstract

tFNA complex rescue acute kidney injury.

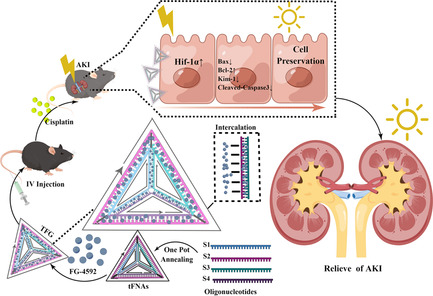

## 1 INTRODUCTION

Cisplatin‐based chemotherapy is used widely to treat head and neck, ovarian, and testicular cancers. It has saved millions of lives since its invention by Rosenberg in 1978.^1^ It is regarded as the ‘Penicillin of Cancer’ due to its unparalleled therapeutic effects, boasting an impressive overall cure rate of 90%.^2^ However, cisplatin chemotherapy can cause cisplatin‐induced acute renal injury (AKI) in cancer patients, and no effective treatments are yet available. Up to 30% of patients experience an immediate loss of renal function and water‐electrolyte imbalance, then face life‐threatening consequences.^3^ Mechanically, the two main metabolic pathways of cisplatin are glomerular filtration and tubular secretion. Cisplatin accumulates in glomerular and tubular and in combination with DNA, forming cisplatin DNA adducts.^4^ After hydration, cisplatin forms an amine‐water complex. This complex replaces the N7 atoms of guanine and adenine in the DNA duplex, forming a DNA adduct that blocks DNA and RNA polymerases to block DNA transcription.^5^, ^6^ The impact of this clinical complication on patients' antitumor treatment and clinical prognosis cannot be overstated. Consequently, developing a chemoprotective agent to mitigate cisplatin‐induced nephrotoxicity is of paramount importance in oncology.^7^, ^8^

Hypoxia inducible factor‐1α (Hif‐1α) as a key factor in tumour metabolic pathway, it can increase the anti‐apoptosis by promoting aerobic glycolysis.^9^ But for normal cells, the upregulation of Hif‐1α can promote tissue regeneration and wound healing.^10^ Activation of Hif‐1α can promote erythropoiesis and oxygen transport. The positive role of Hif‐1α inducer in tissue regeneration has been widely supported by increasing research. FG‐4592, a Hif‐1α inducer, can simulate hypoxic conditions at high altitudes, thereby increasing the production of endogenous erythropoietin (EPO), suggesting its potential application in tissue regeneration and wound healing. In addition, oral FG‐4592 was superior to parenteral epoetin‐α for anaemia in clinical experiments.^11^ It is often administered orally for the treatment of renal anaemia. Emerging studies have reported its ability to promote renal tissue injury healing. Researchers reported that FG‐4592 can prevent renal injury caused by chemotherapy or the ischemia/reperfusion (I/R) process in animal models.^12^, ^13^ These findings imply that FG‐4592 is a promising therapeutic agent in treating cisplatin‐induced renal dysfunction.^12^, ^13^ Due to its Hif‐1α stability and convenient oral administration, it was launched in China in 2018.^14^ FG‐4592 is a prolyl hydroxylase (PHD) inhibitor, an enzyme responsible for hydroxylating Hif‐1α. This hydroxylation process leads to the ubiquitination and subsequent degradation of Hif‐1α. However, its potential cardiovascular and cerebrovascular risks by activating platelets would also cause uncertainty for patients who plan to use this small‐molecule drug in their treatment prescriptions.^15^ Therefore, developing an efficient delivery system with low toxicity and high kidney aggregation is needed to expand the application of FG‐4592.

Since the invention by Nadrian C. Seeman in 1982, DNA origami has become an emerging interdisciplinary field involving medicine, pharmacy, and even engineering over the past three decades.^16^ The tetrahedral framework nucleic acids (tFNAs) were synthesized using four oligonucleotides according to Watson and Crick's base complementation and pairing theory using a one‐pot annealing method.^17^ Because of its advantageous transfection capabilities and stability, this platform is an appropriate and effective solution for drug delivery applications in the biomedical field.^18^, ^19^, ^20^ As a drug delivery system, tFNAs could associate with small‐molecule drugs via certain mechanisms (such as intercalation or electrostatic bonding), which could improve their hydrophobic properties and strengthen their therapeutic effectiveness.^21^, ^22^ This indicates the possibility of forming a tFNAs‐small molecule complex with both components. Further, previous studies have demonstrated that tFNAs exhibited renal aggregation and low nephrotoxicity after intravenous injection in mice, implying that tFNAs are suitable and effective renal‐targeted delivery systems.^23^ Studies on the possibility of tFNAs as suitable drug delivery systems for renal diseases will be a notable breakthrough for nephrology and therapeutic advancements in chemotherapy protection.^24^, ^25^, ^26^

**SCHEME 1:**
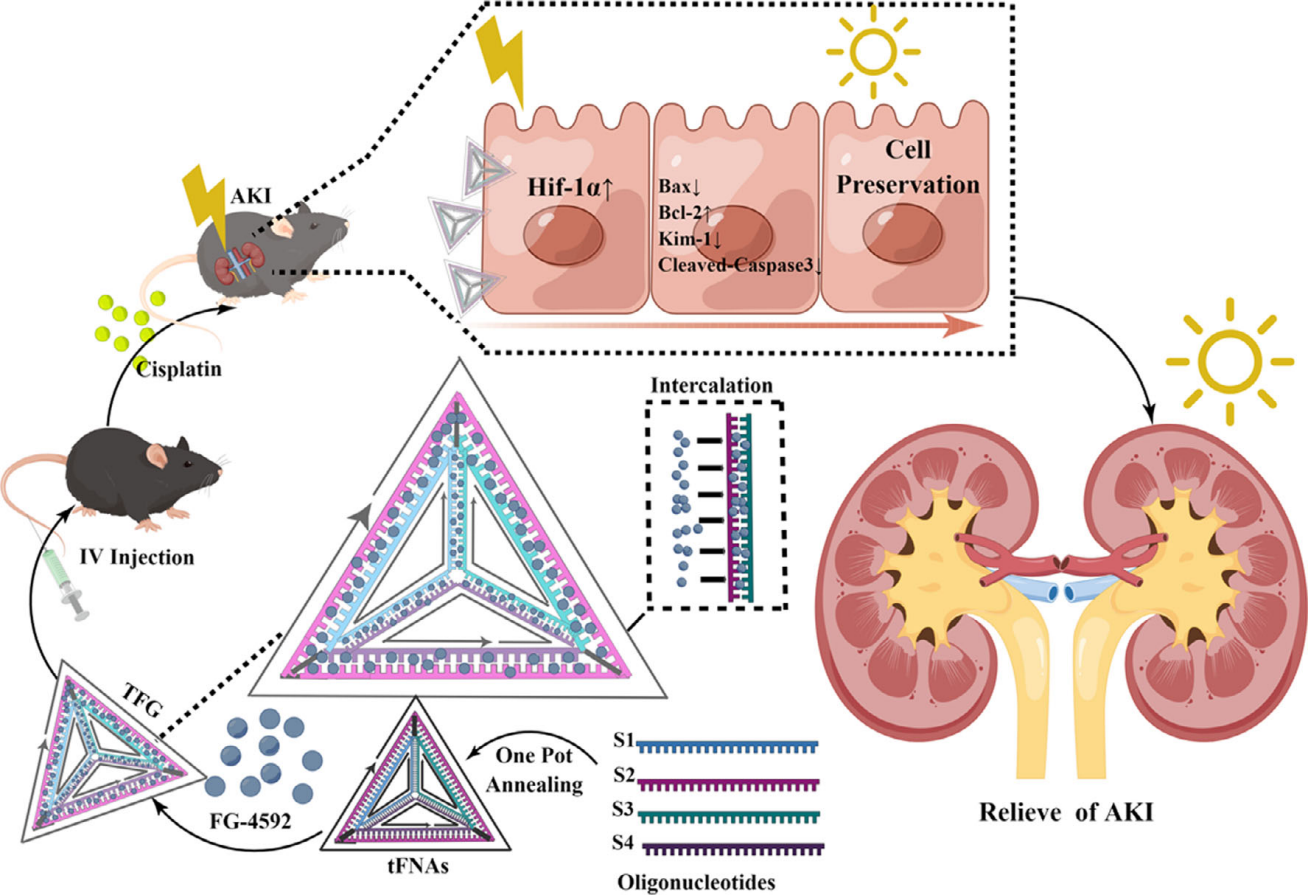
Illustration of the preparation of tFNAs‐FG‐4592 (TFG) complex and its anti‐apoptosis effect on HK‐2 cells to prevent renal tissue from being damaged by cisplatin in animal model.

In this study, we embedded FG‐4592 in tFNAs to form a DNA nanostructure‐small molecule (TFG) complex via intercalation within the DNA structure. Then we applied it to the prevention of cisplatin induced AKI in mice.^27^ The study showed the positive role of the TFG in preventing cisplatin induced cell apoptosis and renal tissue injury in both HK‐2 cell line and animal models. The drug delivery system enhanced the anti‐apoptotic effects of FG‐4592 on mitigating cisplatin‐induced nephrotoxicity and broadening the indications for cisplatin‐based chemotherapy (Scheme S1). By improving the efficacy of cisplatin‐based chemotherapy and minimizing the suffering caused by its side effects, this study contributes to the advancement of humanistic care for patients undergoing chemotherapy and brings attention to the field of chemotherapy protection (Scheme 1).^28^

## 2 MATERIALS AND METHODS

### 2.1 Cell culture

The Human Kidney‐2 (HK‐2) cell line was purchased from BNCC, Henan, China, and was cultured in high glucose DMEM Medium supplemented with 10% (v/w) Fetal Bovine Serum (ZETA, America), 5% (v/w) penicillin/streptomycin solution at 37°C and 5% CO_2_. To maintain optimal growth, the cells should be cultured in fresh medium every 2 days. Subculture the cells at a density of approximately 80% with a 1:2 ratio for each passage.

### 2.2 Preparation of tFNAs and TFG

Four single‐stranded DNAs named S1, S2, S3, S4 were diluted into TM buffer solution, consisting of 50 mM MgCl_2_, and 10 mM Tris–HCl. Then, the pH was adjusted to 8.0. All the components were subjected to a one‐pot annealing program (heated to 95°C for 10 min then cooled at 4°C for 20 min) in PCR after vortexing and centrifugation. The synthesis process followed the protocol described in our previous study, and the oligonucleotide sequences were listed in Table S1. The FG‐4592 was purchased from Wuhan Chemstan Biotechnology and prepared using cell culture‐grade DMSO (MP Biomedicals) as a stock solution. The TFG was prepared by low‐temperature incubation at 4°C for 24 h. We used PAGE to verify the synthesis of the four DNA strands and the successful formation of the DNA‐small molecular complex. To investigate the mechanism of action of DNA and FG‐4592, we performed multiple dilutions of the drug sample with distilled H_2_O (ddH_2_O) and then incubated the samples with 1 μM tFNAs for 24 h. The synthesis was verified using an ultraviolet spectrophotometer at OD260 nm.

### 2.3 Morphology characterization of tFNAs by TEM and AFM

The morphological characteristics of the tFNAs were measured using TEM (Hitachi High Technology, Japan) and AFM (SPM‐9600, Shimadzu Production Institute, Japan). Due to the differences in the experimental process of AFM photography for DNA nanostructures compared to other nanostructures, we have described a detailed experimental process below for reference: For tapping mode imaging in buffer, we utilize short cantilevers on the AFM chip. Sample preparation protocol involves placing 5 μL tFNAs in TAE/Mg or HEPES/Mg on mica, resting it for 0.5–1 min, and adding 25 μL buffer. An extra 30 μL buffer is applied to the tip with the fluid cell oriented upwards. Ensure the alignment of the photodiodes both horizontally and vertically by aligning the laser in ‘AFM & LFM’ and ‘TM AFM’ modes, respectively. Both difference signals should approximate 0. When laser alignment is correct, navigate to the auto tune icon and request a 0.5 V amplitude. The resonance for the small skinny tip should range from 9 to 9.5 kHz.

### 2.4 Cellular uptake of tFNAs using confocal microscopy

As previously described, we assembled tFNAs using the Cy5‐labelled S1 and three other DNA strands (S2, S3, S4). Cells were cultured at a density of 1 × 10^5^ cells/mL in a confocal dish. On the subsequent day, the medium was replaced by 250 nmol/L Cy5‐tFNAs and Cy5‐S1 mixed with serum‐free medium (1 mL per dish). After a 24‐h incubation, the medium was removed and cells were fixed with 4% polyformaldehyde for 25 min. Finally, all the cells were stained with DAPI and a ghost pen cyclic peptide dye (FITC). Each dyeing step lasted for 20–30 min, and the dishes were rinsed thrice with PBS between each step. Finally, all the cells were sealed with 10% glycerol before using a confocal microscope (Olympus Fluoview FV 1000; Tokyo, Japan) to detect the fluorescence signals.

### 2.5 Cell uptake by tFNAs using flow cytometry

Cy5‐S1 and Cy5‐tFNAs were prepared in the dark. Cells were cultured in 12‐well plates. After 48 h, the medium was discarded and the samples were rinsed twice with PBS. To harvest the adherent cells, they were treated with 0.25% trypsin–EDTA (Gibco), detached and then transferred to a 1.5 mL centrifuge tube after being washed twice with PBS at 2000 rpm for 5 min. Finally, cells were suspended in 200 μL of PBS solution before analysis using flow cytometry (CytoFLEX, from Beckman Coulter Inc. located in Brea, CA, USA) with single channel detection at an excitation wavelength of approximately 650 nm.

### 2.6 Living imaging and frozen section of mice to verify renal biodistribution of tFNAs

After 1 week of adaptive breeding, the mice were injected with 2 μM Cy5‐S1 and Cy5‐tFNAs were injected to ensure a dose of 2 μM Cy5 per mouse. 2, 8, 24 h after IV injection, the mice's hearts, livers, spleens, lungs, and kidneys were removed and subjected to in vivo imaging using a Cy5‐detecting instrument. In strict darkness, the collected renal tissues were stored in liquid nitrogen, sliced using a frozen slicer (CM1950; Leica, Germany), and rinsed with PBS. Then, the DAPI was added to the samples following incubation for 5 min. The samples were washed thrice with PBS every 5 min and sealed before observation under a confocal microscope.

### 2.7 Cell viability assay

The CCK8 kit (from MedChemExpress in Monmouth Junction, NJ, USA) was used to evaluate the cytotoxicity of FG‐4592 and TFG and to test their preventive effects on cisplatin‐treated cells. Cells were seeded at 9 × 10^3^ per well in a 96‐well plate following incubation with FG‐4592 or TFG for 24 h. The following day, the medium was replaced with CCK8 solution diluted in a serum‐free medium according to the product description. The samples were tested at an absorbance wavelength of 450 nm for the enzyme marker (Variskan LUX, Thermo Fisher Scientific) to obtain an analysis of cell viability.

### 2.8 In vivo evaluation of biological safety of ssDNA, tFNAs, and TFG

The Balb/C mice were bred under the standard condition for 2 weeks. Then the animals were divided into ssDNA group, tFNAs group and TFG group and every animal was intravenously injected 25 μM of each solution (each solution was diluted into 150 μL 0.9% saline). After 24 h all the animals were sacrificed and the blood were collected in a 1.5 mL Eppendorf tube and centrifuged at the speed of 5000 rpm for 20 min. The plasma was collected and the CREA, UREA level was tested using a blood biochemical analyser.

### 2.9 Quantitative analysis of cell apoptosis using flow cytometry

To perform the Annexin V (FITC)/PI double staining assay, we obtained the staining kit KeyGen Biotech Co, Ltd. Cells were plated at 1.2 × 10^5^/well in a 12‐well plate and treated with serum‐free medium containing 25 μmol/L FG‐4592 and 25 μmol/L TFG for 24 h. The following day, 5 μg/mL cisplatin (Aladdin, Shanghai) in serum‐free medium was added to each well following the incubation for another 24 h. Then we collected cells, washed with PBS, resuspended in 400 μL of 1 × binding buffer, and stained with the two dyes in complete darkness following the product description. Flow cytometry was used for the analysis.

### 2.10 Western blotting analysis of Bax expression

Samples were collected and rinsed twice with PBS. The Whole Protein Extraction Kit was used for cell protein extraction. Cells were plated in a 6‐well plate at a density of 2.5 × 10^5^/well and treated with 25 μmol/L TFG, FG‐4592, and 5 μg/mL cisplatin according to the protocol mentioned above. Following centrifugation at 1 × 10^4^ rpm for 15 min, the samples were mixed with 5 × loading buffer, the supernatant was collected. The protein samples were then heated at 100°C for 15 min, and SDS PAGE gel electrophoresis was performed. The gels were then transferred to PVDF film and sealed with 5% skim milk. After rinsing with TBST, the film was incubated with anti‐Bax antibodies diluted at a ratio of 1:1000 for 10 h at 4°C. The films were then incubated with a secondary antibody diluted 1:5000 for 1 h at room temperature. The samples were imaged using an ChemiDoc System (Bio‐Rad, California, USA), and ImageJ software was used for semi‐quantification for the protein abundance in the gel.

### 2.11 Immunofluorescence detection of protein expression

Cells were seeded at a density of 1 × 10^5^ cells/dish, and the cell treatment protocol was the same as that for the flow cytometry analysis of apoptosis mentioned above. The antibody was then incubated at 4°C for 10 h at recommended incubation concentration provided by the reagent manufacturer (Hif‐1α: 1:200, Bax: 1:200, Cleaved Caspase3: 1:200, Cell Signaling Technology). On the next day, the cells were washed and incubated with a suitable secondary antibody excited at 594 nm and DAPI stain. The following day, after washing and fixation, goat serum was used for blocking, and PBS was used for rinsing. The samples were observed under a confocal microscope (Olympus Fluoview FV 1000, Tokyo, Japan).

### 2.12 Animals breeding and establishment of cisplatin‐induced AKI model

All animal experiments complied with the approval of the West China Hospital of Stomatology Institutional Review Board (WCHSIRB) (Approval Number: WCHSIRB‐D‐2019‐100). Twenty‐four mice, 12 males, and 12 females, aged 6–8 weeks and weighing 18–22 g, were raised under standard feeding conditions for experimental animals. To establish cisplatin‐induced AKI, the mice were grouped into four experimental groups: (1) Control, (2) Cisplatin (10 mg/kg), (3) Cisplatin (10 mg/kg) + FG‐4592 (25 μmol/L), and (4) Cisplatin (10 mg/kg) + TFG (25 μmol/L). In the control group, mice were intravenously injected with 0.9% NaCl for 2 days and intraperitoneally injected with the same solution for another 2 days. In the cisplatin group, mice were intravenously injected with 0.9% NaCl and intraperitoneally injected with 10 mg/kg cisplatin dilution for 2 days. In the FG‐4592 and TFG groups, all solutions were intravenously injected at a dose of 12.5 mg/kg for 2 days. The mice received cisplatin intraperitoneally for the following 2 days.

### 2.13 Western blotting analysis of renal tissue

Put prepared icy lysate (KeyGen Biotech Co., Ltd., Nanjing, China) into a grinding tube and fresh kidney tissue was thoroughly grounded using a grinder at 4°C. Protein samples were prepared as described above. Following SDS gel electrophoresis, the membrane was sealed with skim milk and washed using TBST. The PVDF films were incubated with a 1:1000 antibody (anti‐β‐actin, anti‐Bax, and anti‐Bcl‐2 were obtained from HUABIO, Hangzhou, Zhejiang; anti‐Kim‐1 was purchased from Abcam, Cambridge, UK; Hif‐1α was purchased from Cell Signaling Technology, Massachusetts, USA) and then with the secondary antibody, as mentioned above. Finally, the protein expression signals of the sample were obtained using an Chemdoc system and semi‐quantified using the ImageJ software.

### 2.14 Immunohistochemical and special staining histological analysis of the AKI mice tissue

All organs were collected from the mice. The kidneys of mice were fixed in 4% polyformaldehyde and then conducted HE, Masson, PAS staining for observation of renal tissue damage. Masson staining is a multi‐staining method that are often used to evaluate renal injury. It simultaneously stains collagen fibres, muscle fibres, and nuclei. Observation indicators include the extent of renal tubulointerstitial fibrosis and glomerular interstitial fibrosis. And PAS staining (Periodic Acid Schiff staining) is utilized to stain glycogen, mucopolysaccharides, and certain proteins in cells and matrices. In the context of renal injury, PAS staining reveals the thickening of the glomerular basement membrane, as well as changes in the capillaries and mesangial area within the glomerulus.

And TUNEL staining and immunohistochemical staining for Hif‐1α, Bax and Cleaved Caspase3 were performed to detect the Hif‐1α signalling pathways.

### 2.15 Encapsulation efficiency of TFG

Then we diluted the prepared FG‐4592 according to the concentration gradient multiple and measured the standard curve under UV spectrophotometer. Add FG‐4592 solution to 1 μM tFNAs solution in molar ratios of 1:160, 1:80, 1:40, and 1:20 μmol/L and incubated at 4°C for 8 h. The following day, each component was added to an ultrafiltration tube and placed in a centrifuge at 5000 rpm for 15 min. The supernatant was then taken and the absorbance was measured at a wavelength of 352 nm. Calculate the encapsulation efficiency of TFG based on the standard curve and encapsulation efficiency calculation formula (Table S2 and Figure S1).

### 2.16 Statistical analysis

The researchers replicated the experiments three times to ensure reproducibility. Statistical significance was analysed with GraphPad Prism 8 software, with results presented as *p < 0.05, **p < 0.01, ***p < 0.001, or non‐significant (ns) at *p* > 0.05. *T* tests were performed for each dataset, and outliers or missing data were removed. Our statistical analysis aimed to ensure the validity and reliability of the results.

## 3 RESULTS AND DISCUSSION

### 3.1 Synthesis and characterization of tFNAs

**FIGURE 1:**
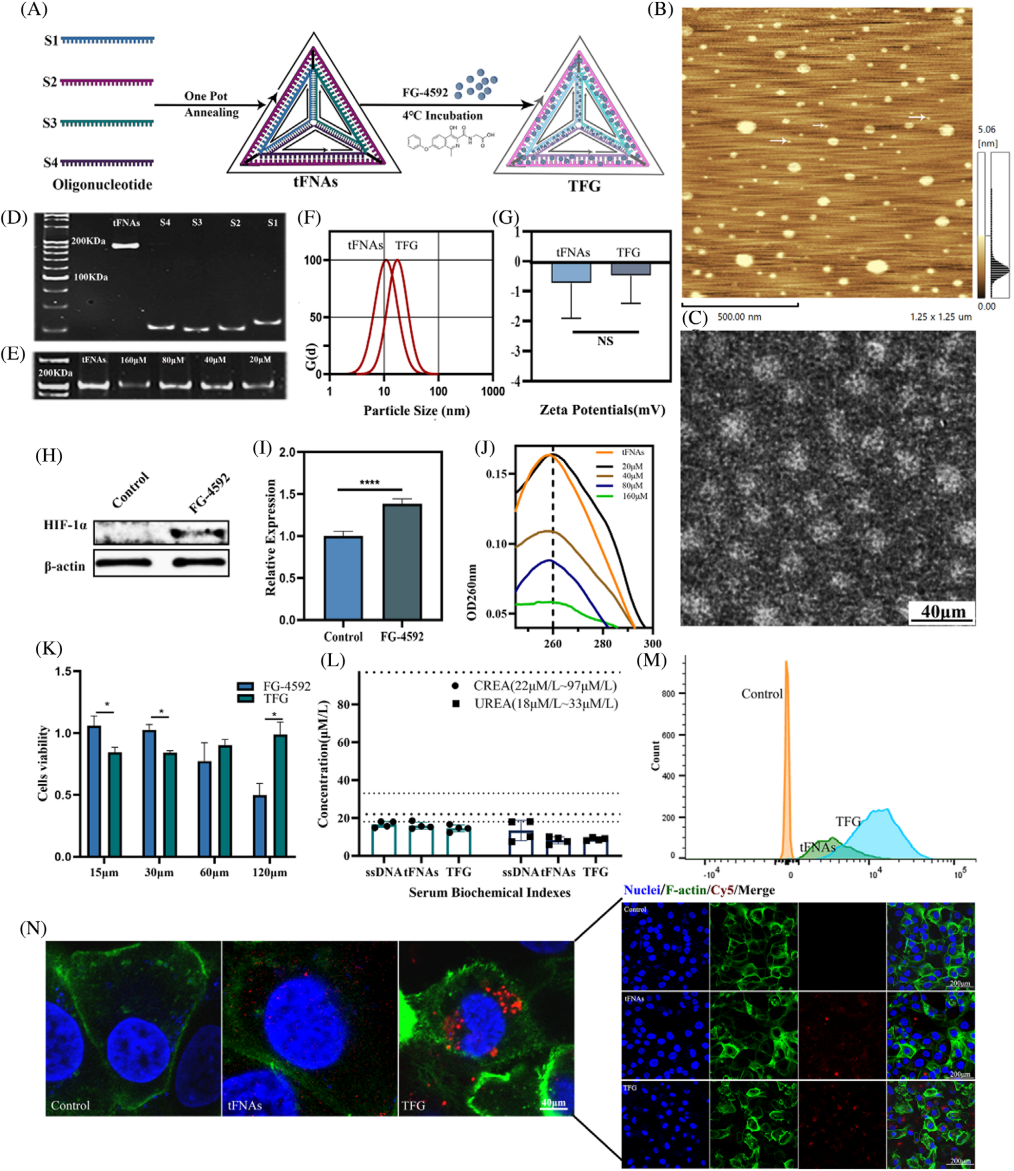
Preparation, characterization, and cell viability analysis of TFG. (A) Preparation pathway of the synthesis of TFG. (B) AFM image of tFNAs, scale bar: 500 nm (C) TEM image of rFNAs, scale bar: 20 nm. (D) PAGE of the synthesis of tFNAs. (E) PAGE of the synthesis of TFG. (F) The particle size of tFNAs and TFG. (G) Zeta potential of tFNAs and TFG. (H) The expression level of Hif‐1α protein was measured by Western Blotting and (I) its semi‐quantification. (J) The UV spectrum of tFNAs and incubated with different concentrations of FG‐4592. (K) Cell viability analysis of FG‐4592 and TFG. (L) The CREA and UREA levels for renal injury of mice after 24 h of tail vein injection of tFNAs and TFG and the dashed line represents the range of normal values. (M) Flow cytometry and (N) cell uptake analysis for Cy5‐tFNAs and Cy5‐TFG after 24 h incubation in HK‐2 cell lines. Cell uptake analysis for tFNAs and TFG after 24 h incubation in HK‐2 cell lines by confocal microscope; statistical analysis: *p < 0.05, **p < 0.01, ***p < 0.001.

A one‐pot annealing method was used to successfully synthesize a novel DNA nanostructure, which we named tFNAs. As shown in Figure 1A, tFNAs were constructed using four specifically designed oligonucleotides (S1, S2, S3, and S4). These oligonucleotides interacted through complementary base pairing, creating a DNA duplex that resulted in a three‐dimensional structure featuring three faces and six edges.^29^, ^30^ The base sequences were listed in Table S1. Molecular weight of the tFNAs was approximately 200 kDa, with individual oligonucleotides contributing approximately 50 kDa to each strand as determined by PAGE (Figure 1D). The excellent repeatability of the experiment suggested that one‐pot annealing is the ideal method for preparing tFNAs. AFM and TEM (Figure 1B,C) analysis demonstrated that tFNAs formed nanoparticles with an approximate size of 23 nm with −0.73 mV Zeta potential and distinct aspect ratios and exhibited triangular shape. As for the different in the particle size between AFM and a Malvern particle analyser measured particle size, the average particle size obtained by AFM (20 nm) was larger than that measured using a Marvin particle analyser (10 nm). This discrepancy might be attributed to potential interactions between the AFM probe and the DNA nanostructure, causing the structure to flatten. Similar challenges were encountered when AFM was used to investigate other morphologies of DNA nanostructure structures. In future investigations, Cryogenic Electron Microscopy (Cryo‐EM) would be a suitable alternative for accurate particle size measurements and morphological analysis.^29^, ^30^

### 3.2 Synthesis and characterization of TFG

The effect of FG‐4592 was first evaluated by Western Blotting in the HK‐2 cells, as presented in Figure 1I,H. FG‐4592 treated group for 24 h showed a notable Hif‐1α‐inducing impact compared with the control group. This was also confirmed by previous studies.^31^ After proving the FG‐4592's ability in inducing Hif‐1α, we then combined then with tFNAs to form TFG. As illustrated in Figure 1E, after incubation with FG‐4592, the signal intensity of tFNAs in the nucleic acid dyes (GelRed) decreased, suggesting a competitive combination with GelRed and validating the successful synthesis of the small‐molecule DNA nanostructure complex.^32^ Moreover, intercalation can cause an achromatic reaction and a redshift in OD260.^30^ As can be shown in ultraviolet spectrophotometry that as the incubation concentration increased, the OD260 values decreased (Figure 1J). We conducted measurements on the hydration particle size and Zeta potential of TFG (Figure 1F,G). When compared to the group without drug loading, TFG showed larger particle size but the change of the particle size was limited. These findings provided direct evidence supporting the interaction between tFNAs and FG‐4592.^33^ This method was simple for nucleic acid drug delivery systems, as designed drugs could be directly loaded through incubation. Hence the fact showed that FG‐4592 is able to combine with tFNAs to form a nanoparticle that are able to act as a drug delivery system for further application in the following experiments.

After successfully synthesizing TFG, we used the CCK8 kit to assess its biosafety. After the fabrication of the TFG, we then tested its cytotoxicity with CCK8 kit and the result showed that compared with FG‐4592 group, the IC_50_ of HK‐2 cells in the TFG group is significantly higher than the FG‐4592 group when the concentration for incubation of FG‐4592 reached 120 μmol/L (Figure 1K). 25 μmol/L of FG‐4592 in both TFG and FG‐4592 groups showed no nephrotoxicity for HK‐2 cells which was selected as our working concentration according to the previous research.^12^ These results suggested that after the fabrication of the TFG, cytotoxicity of FG‐4592 reduced, indicating the improved biocompatibility of TFG. Furthermore, we also conducted in vivo experiment to test the biosafety of TFG by analysing the CREA and UREA level in mice. All the groups showed no damage to the renal tissue in mice indicating a good biocompatibility in the treatment progress. For patients receiving cisplatin basic anti‐tumour therapy, whose physical condition is inferior to that of normal people, TFG exhibited the potential for treating renal side effects caused by chemotherapy for its biocompatible property that is able to perform both curative effect and low toxicity (Figure 1L). In subsequent experiments, we will validate the treatment efficacy following cisplatin modelling.

### 3.3 TFG as a drug delivery system for FG‐4592 showed ideal cell uptake behaviours in HK‐2 cell lines

To verify the ability that TFG could alleviate the cisplatin induced AKI, we first conducted in vitro experiment for its cell uptake ability. Typically, nucleic acids are difficult for cells to take up because of their negative charge and hydrophilicity of nucleic acids.^34^ However, as ssDNA single strands assemble into TFG, the cells could easily take them up (Figure S2). Furthermore, we tested the cell uptake ability of the tFNAs and TFG with confocal microscope and flow cytometry. As in Figure 1N, the fluorescence intensity in the Cy5 channel changed from scattered dots in the tFNAs groups to spots with stronger signal performance in the TFG group. The flow cytometry analysis showed the higher cell uptake than tFNAs group (Figure 1M), consistence with confocal microscope. To understand this issue, we need to conduct more in‐depth research on the mechanisms and signalling pathways related to transport. From the previous research on the tFNAs, this can be attributed to the enhancement of two mechanisms. One is caveolin‐mediated endocytosis. Caveolin is a membrane integrin that exists mainly in vesicles on the cell membrane and serves as a marker protein for cell uptake.^35^ When the nanoparticles reach the cell membrane, the caveolin reaches the plasma membrane by recruiting the binding protein on the cell membrane to form a unique ‘U’ shape, thus promoting their transport into the cytoplasm. The second mechanism involves the rigid structure of TFG, and its nanoparticle size enables it to penetrate different barriers of the human body. But the answer requires further research, we also speculated that this was related to the decrease in nucleic acid negative charge (Figure 1G) and reduced water solubility of small molecule drugs.

By utilizing the high cellular uptake and renal aggregation ability of tFNAs, TFG can not only improve the withdraws of traditional small molecule drugs and enhance their retention ability in vivo, but also minimize the side effects and drug toxicity of drugs in vivo due to their biocompatibility.

### 3.4 TFG showed ideal tissue organizational retention in renal tissue of mice

**FIGURE 2:**
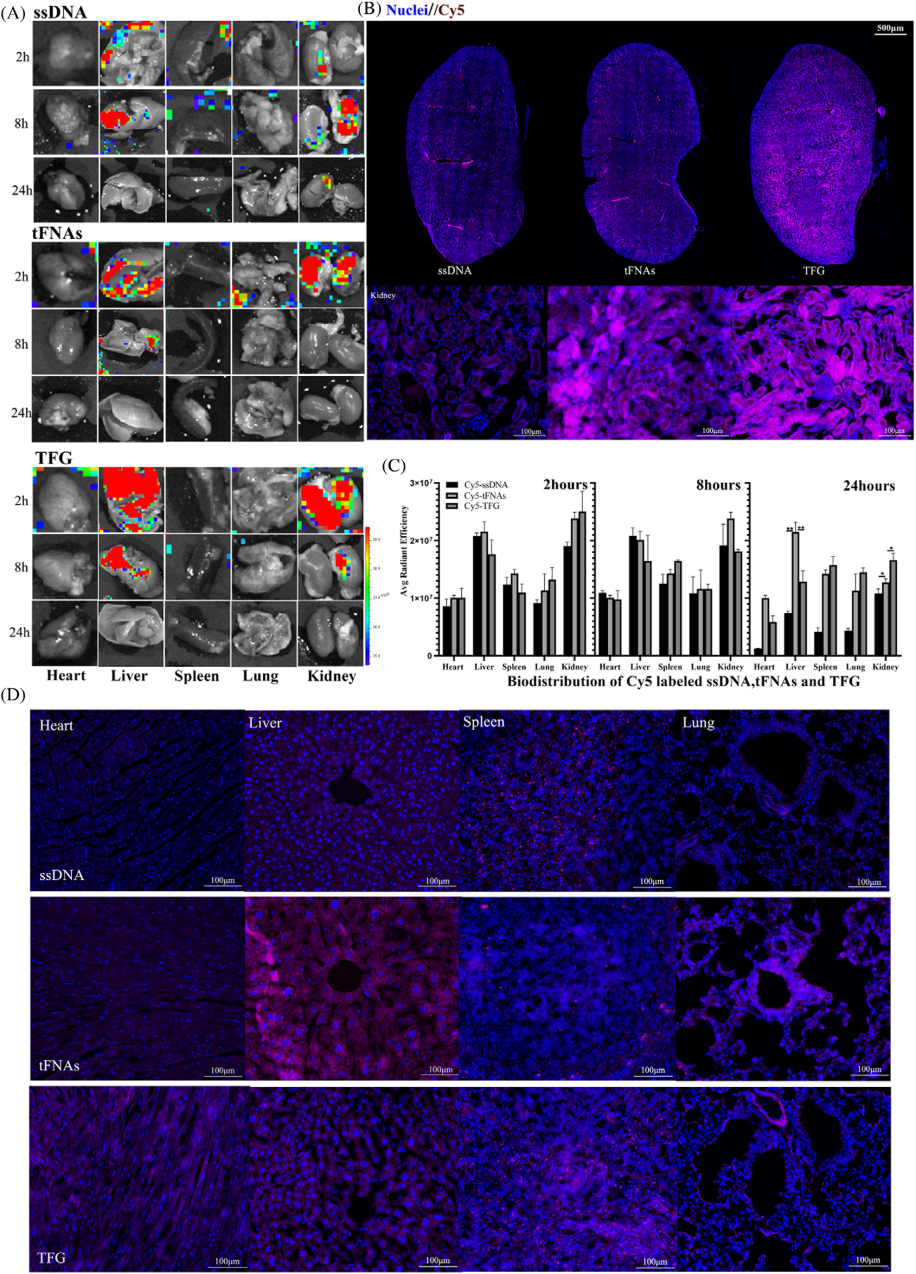
Biodistribution of Cy5 labelled ssDNA, tFNAs and TFG. (A) Ex vivo fluorescent imaging of Heart, Liver, Spleen, Lung and Kidney with intravenous treatments at 2, 8 and 24 h. (B) Fluorescent images of frozen sections of kidney after the injection of ssDNA, tFNAs TFG after 24 h. (C) The semi‐quantification of the mean fluorescence intensity of ssDNA, tFNAs and TFG group at 2 h, 8 h and 24 h after the injection in heart, liver, spleen and lung (*n* = 3). (D) Frozen section of heart, liver, spleen and lung after 24 of the injection. Red represents nanoparticles; blue represents nuclear. Statistical analysis: *p < 0.05, **p < 0.01, ***p < 0.001.

Encouraged by the in vitro experiment results, we then investigated the biodistribution properties of the TFG to explore whether TFG could aggregate in kidney. We detected the in vivo distribution of TFG by measuring the fluorescence intensity of isolated organ tissues at 2, 8, and 24 h after tail vein injection. As shown in Figure 2A, under observation of the living image system compared among all the experiment groups, the kidneys in the TFG group showed the highest fluorescence intensity at 2 h. And even at 24 h, the signals in the kidneys in the TFG group were stronger than those in the liver. Based on our observations in Figure 2B, compared to the control group, we observed large aggregation of Cy5 fluorescence signals in the renal tubules, and TFG group showed higher fluorescence signal than tFNAs group. These results were quite surprising. Typically, nanoparticles ranging in size from 10 to 250 nm are prone to enhanced permeation and retention or can be engulfed by the reticuloendothelial system and absorbed by the liver or spleen.^36^ Instead, the live imaging results showed that the prepared TFG aggregated in the kidneys. We asserted that this phenomenon could not be solely attributed to renal metabolism,^37^ since tFNAs and TFG were identified to be predominantly enriched in the proximal tubules of the renal cortex in cryosections, rather than in the interstitial spaces within the kidney tissue. Furthermore, the semi‐quantitative analysis of each group proved the renal tissue retention of TFG (Figure 2C). Compared with the liver, TFG showed better tissue aggregation in the kidney. The frozen section results also showed the same results in the in vivo imaging of detached organs (Figure 2D). During the 24‐h observation period, the fluorescence signal of TFG showed the longest residence time. The above results demonstrate that TFG could aggregate in the kidneys. Based on the results, we believe that this phenomenon could be attribute to the synergistic effect of renal metabolism and tissue penetration of nanoparticles. These features make nucleic acid nanoparticles an appealing option for researchers to develop tetrahedral‐framework nucleic acid nanostructures to treat renal diseases. Based on the points mentioned above, TFG possesses the potential to reduce the side effects of FG‐4592 and to some extent avoid unnecessary complications caused by drugs.^15^

### 3.5 TFG protected HK‐2 cells by inhibiting apoptotic signalling pathway

**FIGURE 3:**
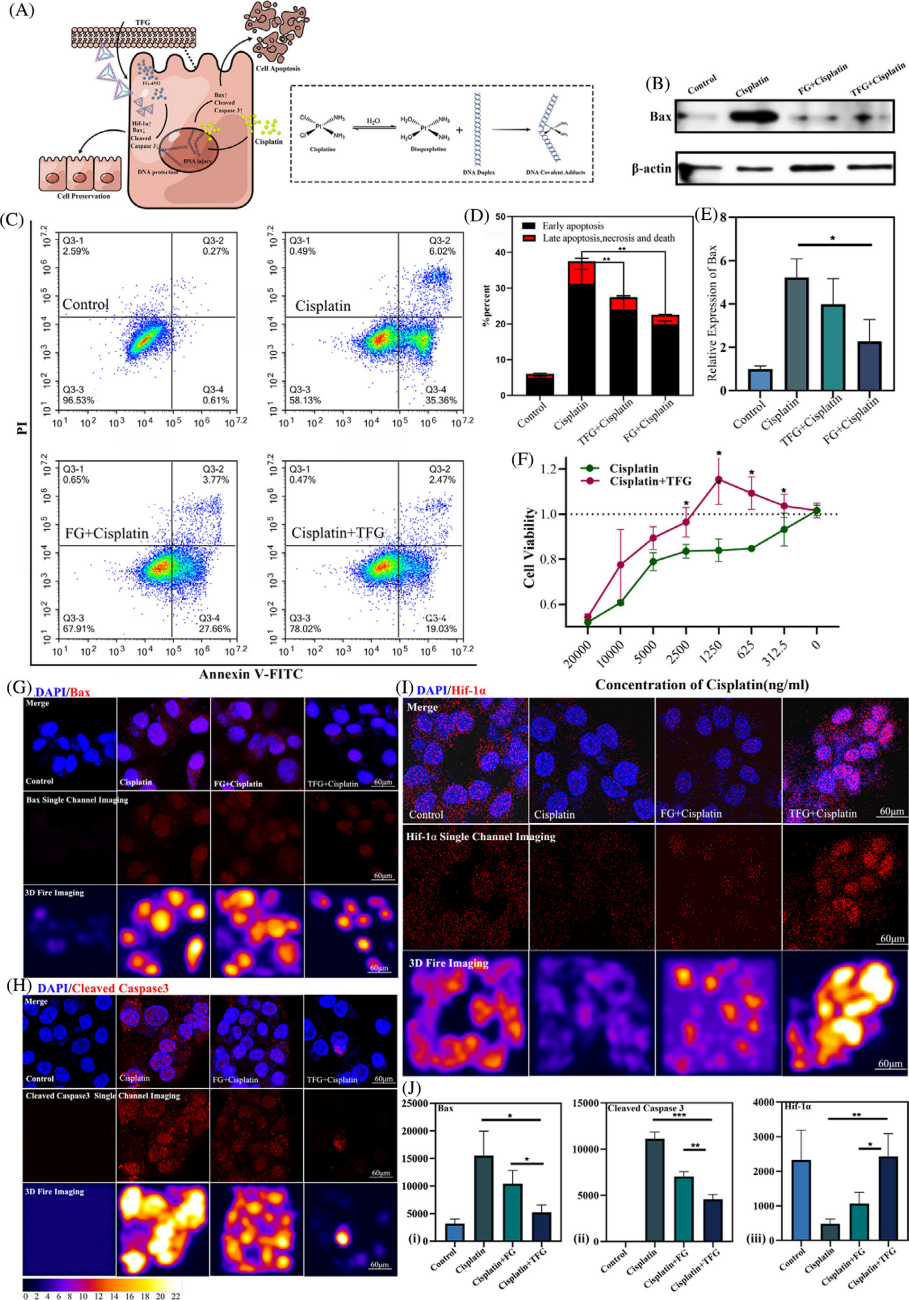
In vitro analysis of the anti‐apoptosis effect of TFG in a cisplatin‐induced HK‐2 cell model. (A) The schematic diagram of cisplatin‐induced nephrotoxicity and pharmacological mechanism of cisplatin induced apoptosis in HK‐2 cells. (B) The expression level of Bax protein was measured by Western Blotting (E) and its semi‐quantification. (C) Quantitative analysis of the anti‐apoptosis effect of TFG by Annexin V(FITC)/PI staining. (D) and the statistical analysis of the trend of early apoptosis and the late apoptosis. (F) Cell viability analysis on cisplatin‐induced HK‐2 cells with and without TFG treatment in the cisplatin induced cell model. (G) Representative confocal images and (J.i) quantitative analysis of Bax in renal tissue. (H) Representative confocal images and (J.ii) quantitative analysis of Cleaved‐Caspase 3 in renal tissue. (I) Representative confocal images and (J.iii) quantitative analysis of Hif‐1α in renal tissue. Statistical analysis: *p < 0.05, **p < 0.01, ***p < 0.001. Statistical analysis: *p < 0.05, **p < 0.01, ***p < 0.001.

Cisplatin performs its antitumor effect via DNA damage caused by coordination with DNA double stranded structure. And for the same reason, the occurrence of cisplatin induced AKI was also based on this mechanism (Figure 3A). In our in vitro study, we used HK‐2 cells, which were frequently used for drug screening, toxicity assessment, and renal disease research.^38^ As a definition of AKI does not exist at the cellular level in vitro experiments, we evaluated the alleviative effect of TFG on cisplatin induced cell apoptosis at the cellular level by Annexin V‐FITC/PI staining method. After statistical analysis of the CCK8 results, we plotted them into a line chart. For trend analysis, we found that TFG with a fixed therapeutic dose could only reduce cell toxicity within a certain range of cisplatin concentrations. As seen from Figure 3F, 25 μM TFG could show an anti‐cytotoxic effect in HK‐2 cells (*p* < 0.05) under the pretreatment of cisplatin in a concentration range between 312.5 ng to 2500 ng. Although the IC_50_ of cisplatin did not differ significantly, this semi‐quantitative experiment showed a trend toward reduced cisplatin cytotoxicity in the presence of TFG. Therefore, for larger doses of cisplatin, higher doses of TFG might be required, but higher doses of TFG were not included in this experiment. Notably, some conclusions drawn in subsequent in vivo experiments might differ from in vitro experiments. Representative images of flow cytometry (Figure 3C) and its quantitative analysis (Figure 3D) showed that TFG reduced early and late apoptosis at 24 h after cisplatin treatment, implying that TFG could reduce the toxicity of cisplatin at the cellular level.^39^ Moreover, we used CCK8 kit to test cell viability after the treatment of TFG in cisplatin induced HK‐2 at different concentration level (Figure 3F).^12^ This is because the renal aggregation performance of TFG, as a nano delivery system, can only be demonstrated in animals. Under the conditions of intravenous injection in animals, the uptake efficiency of FG‐4592 alone was very low.^40^ In terms of the mechanism, since cisplatin triggered cell apoptosis by producing DNA intrachain and chain adducts, we proposed that DNA nanostructure, owing to its DNA nature, was likely to replace normal cells to bind with free cisplatin, thereby reducing adequate cisplatin levels in kidney tissue. However, this assertion required further quantitative experiments for verification. In this study, we did not include naked tFNAs group in the treatment. That was because when the concentration of the tFNAs was higher than 375 nmol/L, it would lose any biological activity and did not cause toxicity to cells. Hence, in this study the 1000 nmol/L concentration of TFG would not cause any effect.^41^ What is more, in this experiment, we focused on the delivery effect of tFNAs, and chose Hif‐1α inducing agents, utilizing the kidney aggregation property of the tFNAs to deliver the Hif‐1α inducer to kidney and analysed their potential for renal drug delivery through mechanism validation. Obviously, our data could cover all nodes of our validation process. Two sets of immunofluorescence images and relative expression analysis of Bax also showed that TFG reduced the apoptotic effect of cisplatin (*p* < 0.05).

Given that FG‐4592 exerts its effects by upregulating Hif‐1α, we first explored the expression of cellular Hif‐1α in the TFG treatment group with or without cisplatin‐induced modelling. We firstly observed the increased expression Hif‐1α after cisplatin treatment in the TFG group which indicated the successful delivery of the FG‐4592 molecule (Figure 3I,J.iii). The results showed that TFG could upregulate Hif‐1α and then downregulate apoptosis signalling pathways. Bcl‐2 and Bax are the two main factors that affect cell apoptosis. Bax, a pro‐apoptotic protein, channels lipid membranes at both neutral and acidic pH. And Bcl‐2 blocks this process. Western Blotting results indicated that the Bax expression in the TFG and FG‐4592 groups was significantly reduced compared to the cisplatin‐treated group (Figure 3B,E). Moreover, the expression levels of Cleaved‐Caspase 3 and Bax were also assessed in confocal microscopy experiments (Figure 3G,H,J). It was worth noting that we did not include Bcl‐2 in our in vitro experimental design. This was because Bcl‐2 is not expressed in HK‐2 cell lines. Our research confirmed that the TFG complex could transform FG‐4592 to the cell's interior, where it performed its distinct function of upregulating Hif‐1α (*p* < 0.01). To sum up, in vitro experiments proved that TFG could protect HK‐2 cells by inhibiting apoptotic signalling pathways.

### 3.6 TFG alleviated acute kidney injury induced by cisplatin

**FIGURE 4:**
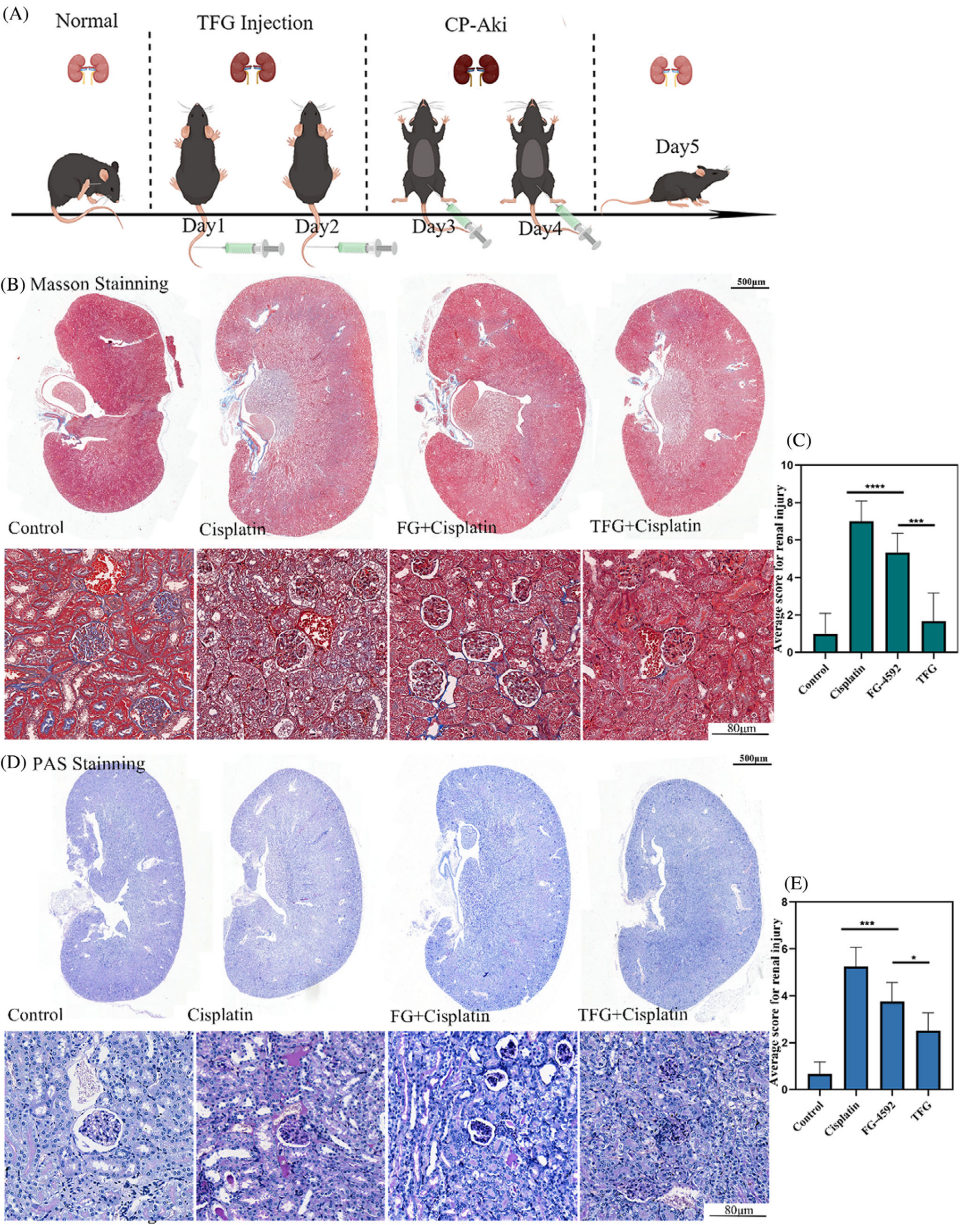
Histological analysis of the cisplatin‐induced AKI animals after treatment of TFG. (A) The schematic diagram showing the method of cisplatin induced AKI and the treatment strategy. (B) Masson staining of kidney tissue and (C) semi‐quantification analysis. (D) PAS staining and (E) semi‐quantification. Statistical analysis: *p < 0.05, **p < 0.01, ***p < 0.001. *n* = 3.

Due to the positive effect in the anti‐apoptotic effect in cisplatin treated HK‐2 cells, we further evaluated the effect of TFG on cisplatin induced renal injury in vivo. In our study, we administered a double injection (10 mg/kg/day) of cisplatin to induce AKI in mice. Compared to a single injection (20 mg/kg/day),^42^ the double injection method could cause stable AKI in mice and ensure their survival. As shown in Figure 4A, we performed an IV injection of 25 μM TFG into the mice at the first 2 days, and then the double injection of cisplatin at Day 3 and Day 4. The TFG dosage was selected on a study by Zhang.^12^ At Day 5, mice were sacrificed and the kidneys were collected. Then renal histological analysis (Masson, H&E and PAS), and semi‐quantitative analysis were performed using a tubular scoring system.^43^ Masson staining of renal tissue sections revealed new vessels around the glomerulus in the FG and TFG groups (Figure 4B,C), suggesting the TFG complex significantly alleviated cisplatin‐induced renal tubular injury in the tubular cells. This finding was consistent with the pharmacological mechanism of FG‐4592, which upregulated VEGF signalling pathways in a compensatory lung growth model. Furthermore, PAS staining of renal tissue sections revealed that the TFG caused less glycogen deposition and the thickening of the glomerular basement membrane (Figure 4D,E). This phenomenon was attributed to the permeation and renal aggregation of TFG. And owing to the low solubility and low bioavailability of FG4592, its effectiveness is far inferior to TFG.^44^

**FIGURE 5:**
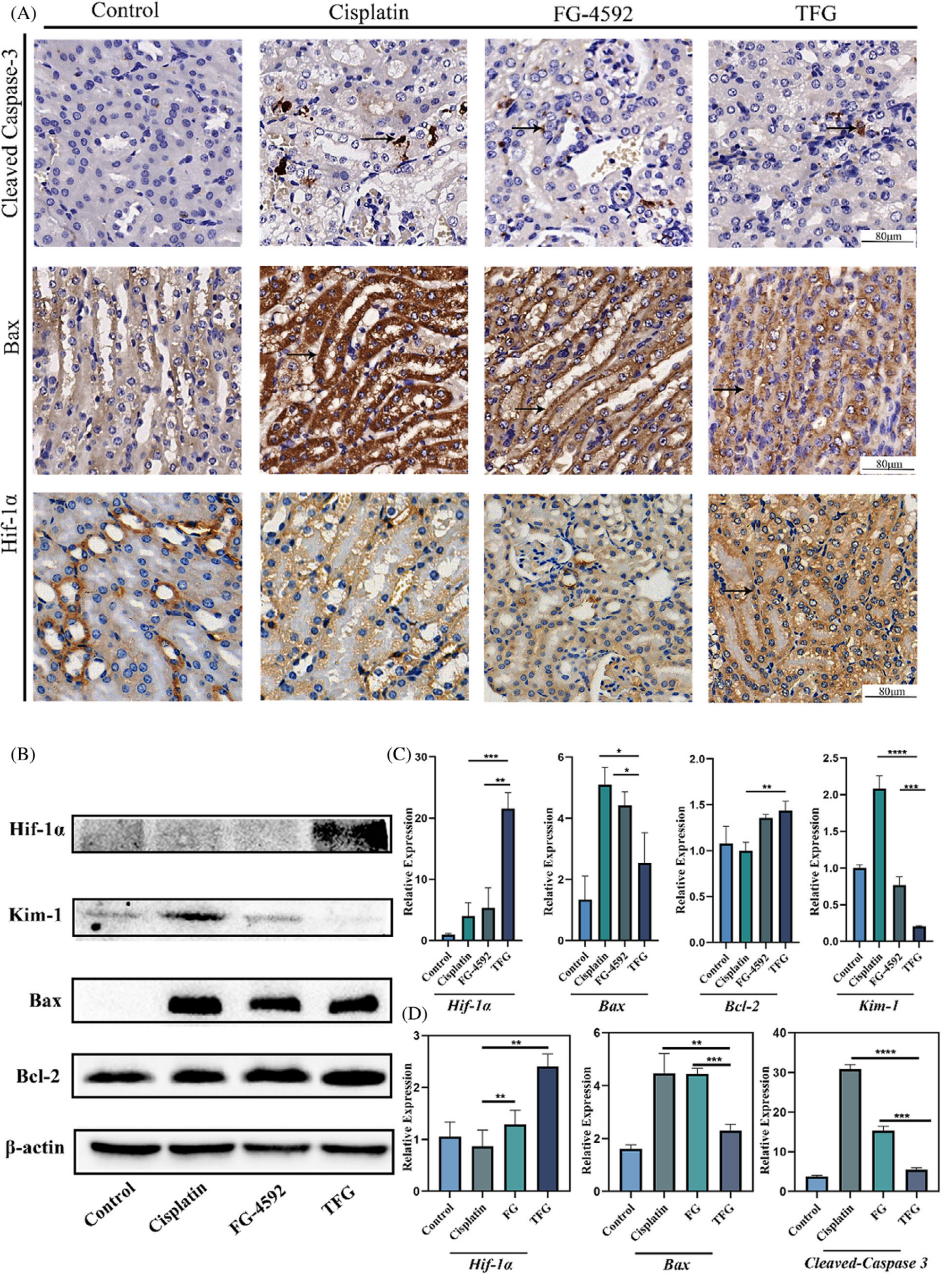
The mechanism of the anti‐apoptosis property of TFG and its Hif‐1α inducing effect. (A) Immunohistochemical analysis of Cleaved‐Caspase 3, Bax and Hif‐1α and (D) semi‐quantification analysis. (B) Western Blotting analysis of the expression of Hif‐1α, Bcl‐2, Bax and Kim‐1 and (C) semi‐quantification. Control, Cisplatin, FG + Cisplatin, and TFG + Cisplatin groups as the Control, Cisplatin, FG, and TFG groups, respectively. Statistical analysis: *p < 0.05, **p < 0.01, ***p < 0.001.

To further verify the signalling pathway of the preventative effect of TFG, we have measured the expression of Hif‐1α, cell apoptosis‐related protein (Bax, Bcl‐2 and Cleaved Caspase‐3), and renal injury‐related markers Kim‐1 in the kidney homogenate of the mice. As shown in Figure 5A, immunohistochemical experiments showed that TFG group could significantly upregulate the expression of Hif‐1α in the tubular cells, and downregulate the apoptosis‐related factors Bax and Cleaved Caspase‐3 (Figure 5A), compared with Cisplatin group. Additionally, western blotting assay also confirmed that the Bax was downregulated in the TFG group, indicating the relieve of the cell apoptosis of the kidney tissue in Figure 5B. And the Kim‐1 was downregulated in the TFG as well, indicating the relieve of the renal injury. Kim‐1 factor, a transmembrane glycoprotein from renal proximal tubular epithelial cells, which was upregulated when kidney injury occurs (Figure 5C).^45^ Above all, the ability that TFG could relieve renal injury induced by cisplatin be caused by the upregulation of the Hif‐1α. And it inhibited the expression of apoptosis related proteins Bax and Cleaved Caspase‐3. All the data was collected and analysed using statistical method (*n* = 3), and the Figure 5D. But the inner mechanism of the relationship between Hif‐1α and the cell apoptosis‐related factors is unknown and needs further study and more solid evidence. In addition, the anti‐injury effect was also shown in TUNEL staining and HE staining of kidney (Figures S3 and S4). Taken together, the TFG were able to perform anti apoptotic effect to prevent the kidney from being damaged by cisplatin by utilizing the kidney aggregation effect of the TFG. But whether this complex is able to alleviate kidney damage caused by other toxic substances further exploration is needed.^46^, ^47^, ^48^

## 4 CONCLUSION

In conclusion, we successfully synthesized a DNA nanostructure complex, the TFG, using FG‐4592 and tFNAs. This complex demonstrated renal aggregation, superior tissue and cellular permeability, and exceptional biocompatibility, suggesting its potential as a drug delivery platform for treating renal disease. After intercalation, TFG exhibited the unique capacity to attenuate cisplatin‐induced nephrotoxicity in HK‐2 cell lines and mice by inhibiting apoptotic signalling pathways. TFG‐treated mice showed significant relieve in renal tissue. This phenomenon was attributed to the upregulation of Hif‐1α facilitated by FG‐4592, which suppressed apoptosis signalling pathways. This study contributes to the field in terms of two critical aspects, namely, the development of chemotherapy protection strategies and a thorough investigation of the exceptional biocompatibility of DNA nanostructure in renal disease treatment. Hence, this small‐molecule drug appears to possess the ability to promote tissue regeneration, and some researchers have begun to study its role in wound and bone healing.^49^, ^50^

## AUTHOR CONTRIBUTIONS

Yuanchong Chen and Jiangshan Xu perform the expertiments, Sirong Shi, Wenjuan Ma, Weitong Cui, and Ran Yan analyse the data, Yunfeng Lin supervise the project.

## CONFLICT OF INTEREST STATEMENT

The authors have no conflict of interest to declare.

## DECLARATION OF GENERATIVE AI AND AI‐ASSISTED TECHNOLOGIES IN THE WRITING PROCESS

During the preparation of this work, the author did not use any Generative AI and AI‐assisted technologies in the writing process.

## Supporting information


**Data S1.** Supporting Information

## Data Availability

The data that support the findings of this study are available from the corresponding author upon reasonable request.
