# Supplementary material for: A DNA nanostructure‐Hif‐1α inducer complex as novel nanotherapy against cisplatin‐induced acute kidney injury
Source: Cell Prolif. 2024 Jan 14;57(6):e13601. doi: 10.1111/cpr.13601 (PMC11150135; doi:10.1111/cpr.13601)
Supplement: Supplementary file 1 — Data S1. Supporting Information [file CPR-57-e13601-s001.docx]

*Supporting Information*

A DNA Nanostructure-Hif-1α Inducer Complex as Novel Nanotherapy Against Cisplatin-Induced Acute Kidney Injury

Yuanchong Chen^1, 2^, Jiangshan Xu^1, 2^, Sirong Shi^1, 2^, Wenjuan Ma^1, 2^, Weitong Cui^1, 2^, Ran Yan^1, 2^, Yunfeng Lin^1, 2^*

^1^ State Key Laboratory of Oral Diseases, National Center for Stomatology, National Clinical Research Center for Oral Diseases, West China Hospital of Stomatology, Sichuan University, Chengdu, Sichuan 610041, China

^2^ Sichuan Provincial Engineering Research Center of Oral Biomaterials, Chengdu, Sichuan 610041, China

*Corresponding authors: Yunfeng Lin

*Yunfeng Lin: E-mail: yunfenglin@scu.edu.cn

State Key Laboratory of Oral Diseases, National Center for Stomatology, National Clinical Research Center for Oral Diseases, West China Hospital of Stomatology, Sichuan University, Chengdu 610041, P. R. China

**Table S1.** The Sequence of Each DNA Strand Involved in the Study

| **Strand** | **Sequence** |  |
| --- | --- | --- |
| **S1** | **5′- ATTTATCACCCGCCATAGTAGACGTATCACCAGGCAGTTGAGACGAACATTCCTAAGTCTGAA-3′** |  |
| **S2** | **5′-ACATGCGAGGGTCCAATACCGACGATTACAGCTTGCTACACGATTCAGACTTAGGAATGTTCG-3′** |  |
| **S3** | **5′-ACTACTATGGCGGGTGATAAAACGTGTAGCAAGCTGTAATCGACGGGAAGAGCATGCCCATCC-3′** |  |
| **S4** | **5′-ACGGTATTGGACCCTCGCATGACTCAACTGCCTGGTGATACGAGGATGGGCATGCTCTTCCCG-3′** |  |
| **S1-Cy5** | **5′Cy5-ATTTATCACCCGCCATAGTAGACGTATCACCAGGCAGTTGAGACGAACATTCCTAAGTCTGAA-3′** |  |

**Table S2**. Encapsulation Efficiency of TFG

| Concentration (μM/L) | \| 20 \| 40 \| 80 \| 160 \| \| --- \| --- \| --- \| --- \| | |  |
| --- | --- | --- | --- | --- | --- | --- | --- |
| Average Encapsulation Efficiency (%) | | 11.49 13.74 22.81 7.6 | |
| SD | 0.03 0.06 0.02 0.03 | |  |

$$EE\%=\frac{Encapsuled Fg4592}{Total FG4592}\times100\%$$

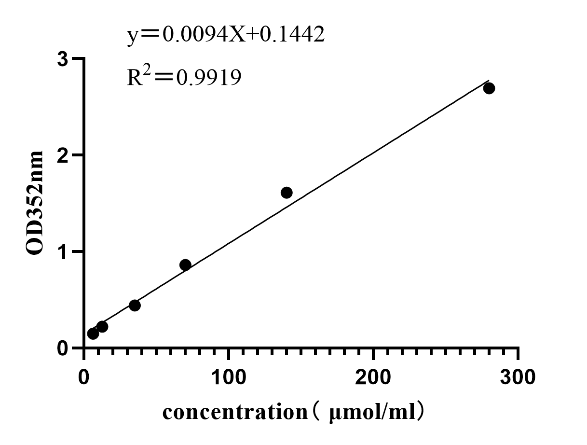


**Fig. S1.** The standard curve of the FG-4592 between the concentration range from 0 to 300.

**
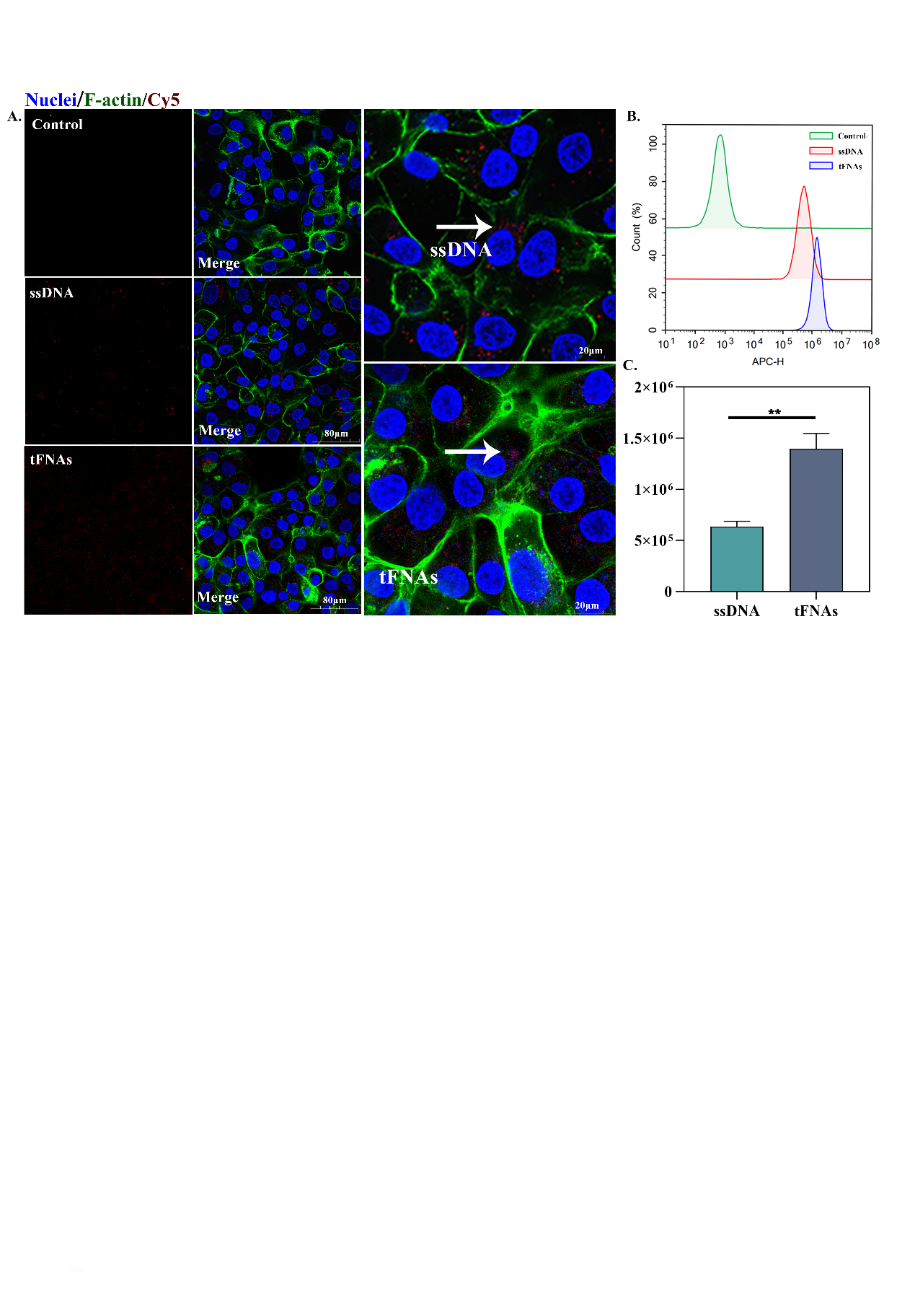
**

**Fig. S2.** (A) represent the cell intake ability of the tFNAs and ssDNAs after the incubation of each nucleic after 24 hours.S2(B) represent the cell intake behavior of tFNAs and ssDNA by using flow cytometry.S2 (C) represent is the statistical analysis of the cytometry results and the T test was used for evaluating the cell intake ability of each group (n=3).P＜0.05


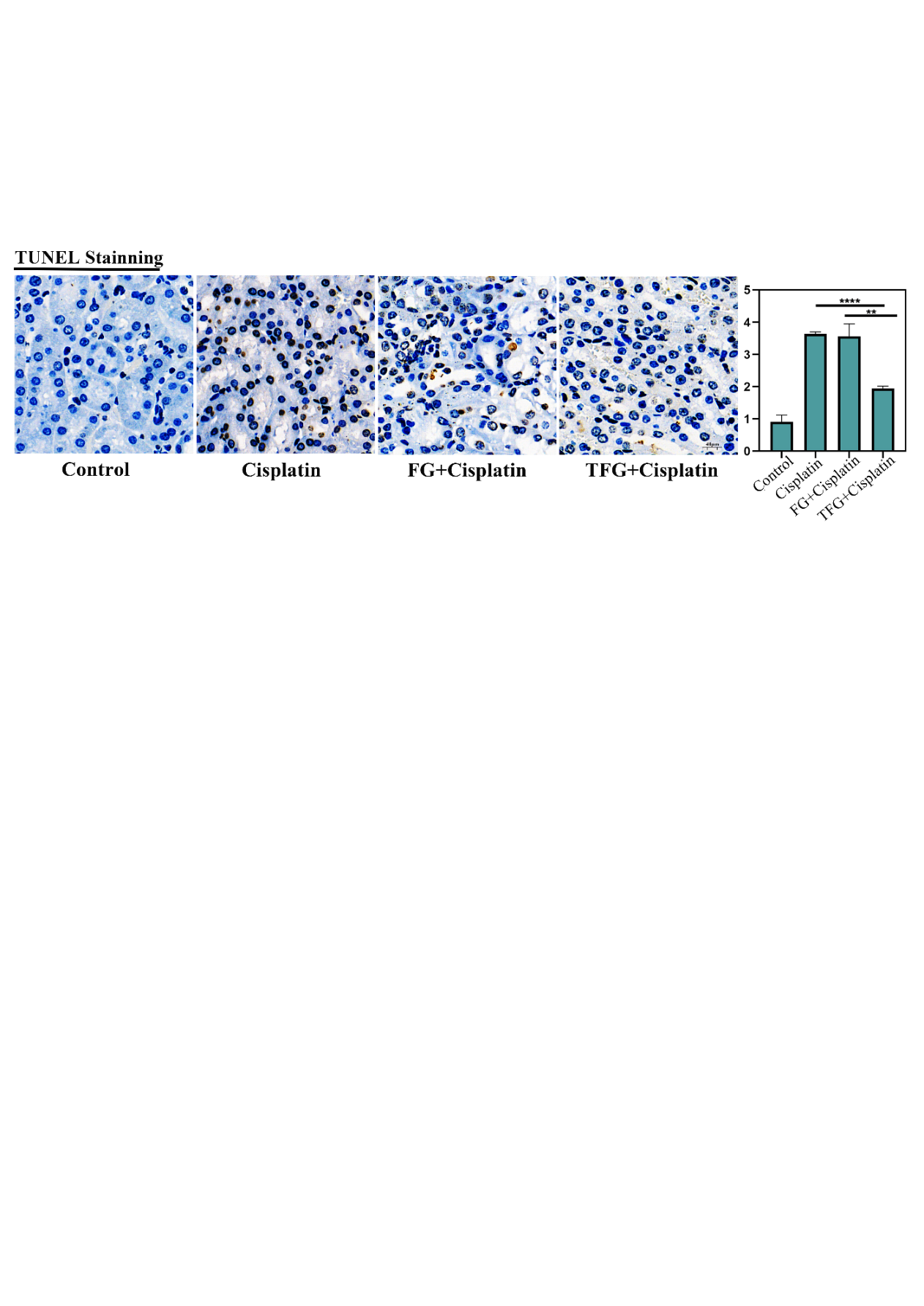


**Fig. S3.** The TUNEL staining of the kidney tissue of the cisplatin induced renal injury models observing in the microscope and the statistical analysis of the result by calculating apoptosis cell using image J (n=3). P＜0.05.


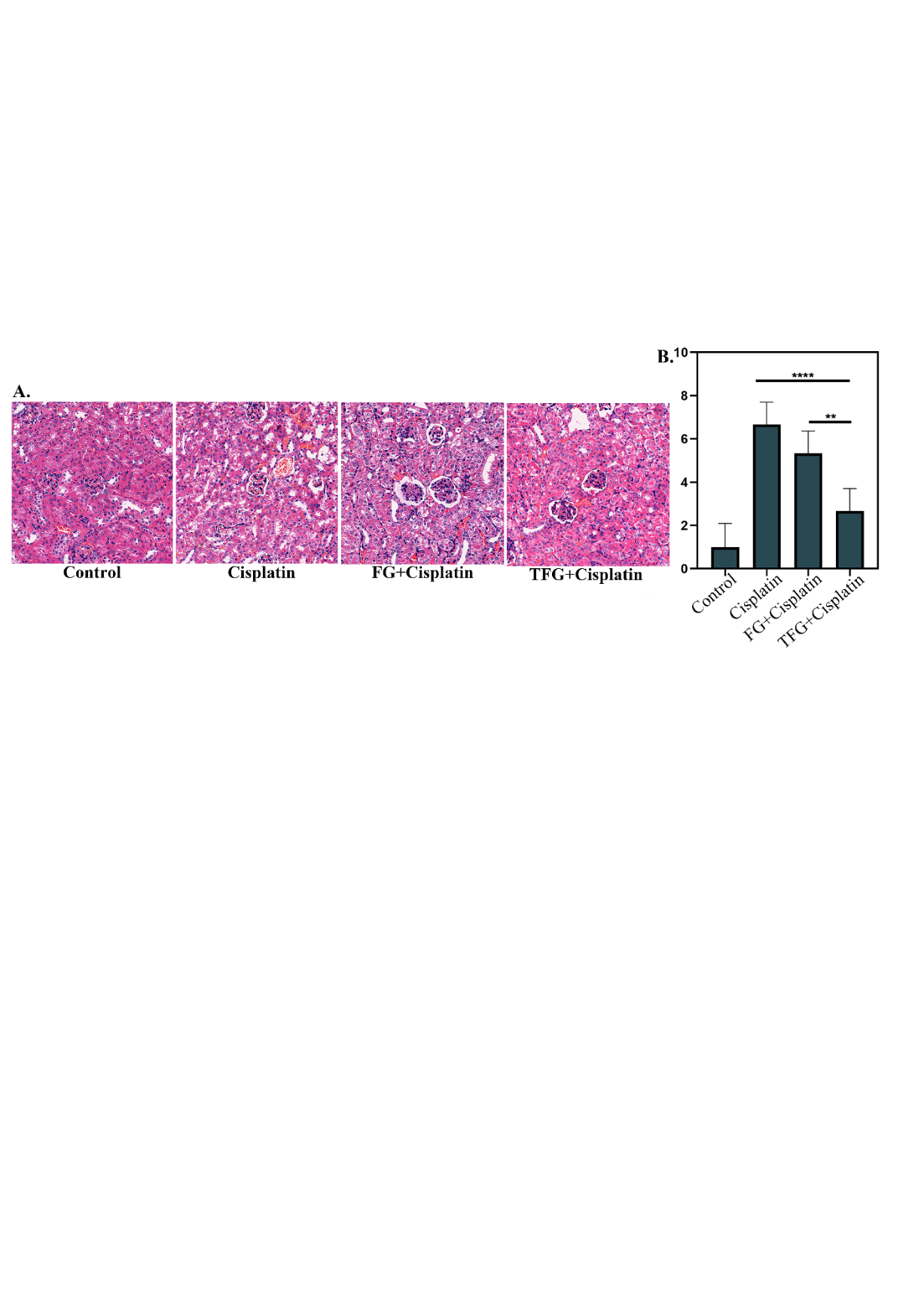


**Fig. S4.** The HE staining of the kidney tissue of the cisplatin induced renal injury models observing in the microscope and the statistical analysis of the result by calculating apoptosis cell using image J (n=3). P＜0.05.


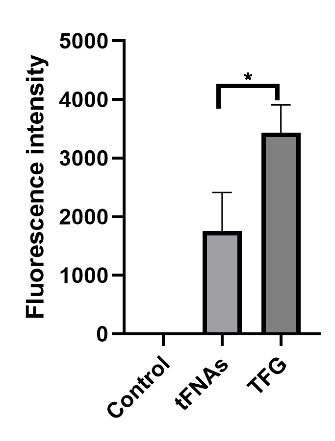


**Fig. S5.** The statistical analysis for the cell uptake ability of the tFNAs and TFG under the observation of the confocal microscope. The T test was used for calculating the statistical significant of each group (n=3). P＜0.05.
